# From fundamental understanding to engineering of carboxysomes for biotechnological applications

**DOI:** 10.1016/j.xplc.2026.101882

**Published:** 2026-04-30

**Authors:** Tianpei Li, Weixian Chen, Vincent Chriscoli, Lu-Ning Liu

**Affiliations:** 1 Institute of Systems, Molecular and Integrative Biology, University of Liverpool, Liverpool L69 7ZB, UK; 2 MOE Key Laboratory of Evolution and Marine Biodiversity, Frontiers Science Center for Deep Ocean Multispheres and Earth System & College of Marine Life Sciences, Ocean University of China, Qingdao 266003, China

**Keywords:** carboxysome, carbon fixation, enzyme encapsulation, permeability, self-assembly, bioengineering

## Abstract

Carboxysomes are self-assembling proteinaceous microcompartments that encapsulate ribulose-1,5-bisphosphate carboxylase/oxygenase (Rubisco) and carbonic anhydrase within a semipermeable shell, thereby increasing local CO_2_ concentrations around Rubisco to improve carbon fixation. Their inherent design principles, including programmable architecture, cargo encapsulation, semi-permeability, and modular assembly, position carboxysomes as powerful models in synthetic biology and bioengineering, offering unprecedented opportunities to increase carbon assimilation and unlock novel biotechnological functions. This review summarizes our current understanding of the molecular mechanisms underlying carboxysome structure, assembly, and function, highlighting recent breakthroughs and key challenges such as achieving precise control of shell permeability, efficient cargo encapsulation, and integration of carboxysomes into heterologous hosts. We also outline emerging strategies and future perspectives for engineering carboxysomes as enhanced CO_2_-fixing engines and repurposing them as versatile nanomaterials for biotechnological applications. Together, these advances underscore the growing potential of carboxysome engineering to transform carbon-fixation pathways across diverse biological systems.

## Teaser

Carboxysomes are protein-based microcompartments that enhance CO_2_ fixation by concentrating CO_2_ around Rubisco through coordinated shell architecture, cargo encapsulation, and selective permeability. This review synthesizes current understanding of carboxysome structure, assembly, and function, while highlighting recent advances, key challenges, and emerging engineering strategies. Together, these insights underscore the growing potential of carboxysomes as modular platforms for improving carbon assimilation and enabling new biotechnological applications.

## What are carboxysomes?

Bacterial microcompartments (BMCs) are a diverse family of protein-based organelles found across 45 bacterial phyla (Cabello-Yeves et al., 2022). They consist of selectively permeable proteinaceous shells that enclose enzymes, thereby creating specialized microenvironments that support spatially segregated catalytic reactions in the cytoplasm. The permeability and encapsulation properties of BMCs enable bacteria to optimize metabolic pathways by concentrating enzymes and substrates inside the compartment, enhancing substrate channeling, and sequestering toxic or volatile intermediates (Kerfeld et al., 2018). Different classes of BMCs support a wide range of metabolic processes, from carbon fixation in autotrophs (carboxysomes, CBs) to the catabolism of small organic compounds in pathogenic bacteria (metabolosomes) (Liu, 2021). CBs are among the best-characterized and widespread BMCs, serving as the primary CO_2_-fixing organelles in all cyanobacteria and some chemoautotrophic bacteria (Liu, 2022).

**Figure 1:**
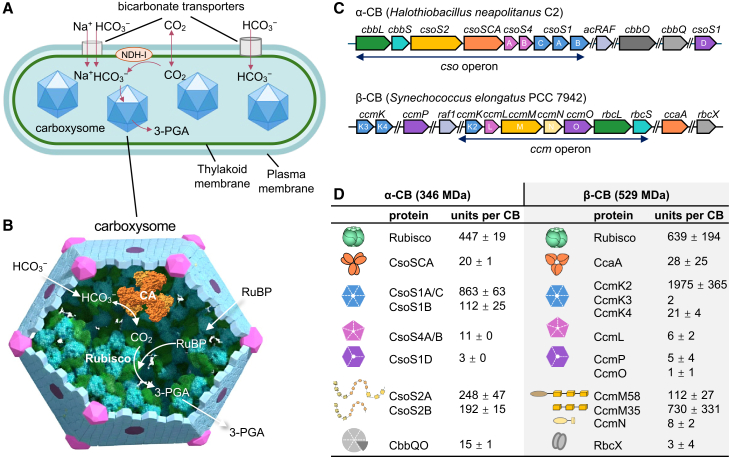
Overview of the function and structural organization of CBs. **(A)** The cyanobacterial CO_2_-concentrating mechanism (CCM), which includes CBs and inorganic carbon–pumping systems, increases intracellular inorganic carbon levels and enhances carbon fixation. Membrane-bound bicarbonate transporters mediate HCO_3_^−^ uptake, and NDH-I complexes in the thylakoid membrane convert CO_2_, which enters the cell by passive diffusion, into HCO_3_^−^, thereby increasing the intracellular HCO_3_^−^ concentration available to CBs. **(B–D)(B)** The CB encapsulates the CO_2_-fixing enzyme Rubisco and carbonic anhydrase (CA) within a semipermeable icosahedral protein shell. Accumulated bicarbonate diffuses across the shell and is converted to CO_2_ by CA inside the CB. The shell allows the passage of ribulose-1,5-bisphosphate (RuBP) and HCO_3_^−^ into the CB while restricting CO_2_ leakage, thereby maintaining high levels of CO_2_ around Rubisco and enhancing its carboxylation efficiency. The gene organization **(C)** and protein stoichiometry **(D)** of α- and β-CBs in two model organisms, *Halothiobacillus neapolitanus* C2 and *Synechococcus elongatus* PCC 7942, are shown. Genes encoding Rubisco large and small subunits are shown in green and cyan, respectively; CA in red-orange; linker proteins in orange or yellow; pentameric proteins in magenta; hexameric proteins in blue; trimeric proteins in purple; and Rubisco activases and chaperones in gray.

CBs, together with inorganic carbon transporters that actively accumulate bicarbonate in the cytosol, constitute the core of the bacterial CO_2_-concentrating mechanism (CCM) (Figure 1A). The CCM evolved in many autotrophic bacteria as an adaptation to low ambient CO_2_ levels (Nguyen et al., 2025a). By increasing the local CO_2_ concentration within CBs around ribulose-1,5-bisphosphate carboxylase/oxygenase (Rubisco), which exhibits intrinsically low CO_2_ affinity and low catalytic turnover rates (de Pins et al., 2025), the CCM enhances the efficiency of carbon fixation and supports bacterial growth under CO_2_-limited conditions. CBs encapsulate Rubisco and carbonic anhydrase (CA) within a polyhedral proteinaceous shell. This semi-permeable shell permits the entry of HCO₃⁻, which is rapidly converted to CO₂ by internal CA, resulting in a high-CO₂ microenvironment within the shell that facilitates Rubisco carboxylation and suppresses its oxygenase activity (Figure 1B). The CB shell is composed primarily of three groups of building blocks: hexamers (BMC-Hs) and trimers (BMC-Ts), which form the facets, and pentamers (BMC-Ps), which cap the vertices (Correa et al., 2025). Hundreds of these building blocks can self-assemble into a highly ordered polyhedral shell that sequesters Rubisco and CA, thereby forming complete CBs.

## Natural properties of carboxysomes make them promising candidates for biotechnology

**Figure 2:**
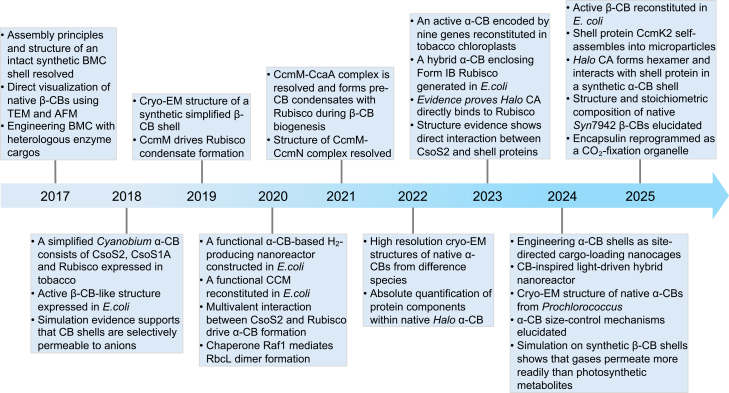
Timeline of major milestones in the study of CB structure, assembly, and bioengineering. CB, carboxysome; AFM, atomic force microscopy; TEM, transmission electron microscopy; *Halo*, *Halothiobacillus neapolitanus* C2; *Syn*7942, *Synechococcus elongatus* PCC 7942; *E. coli*, *Escherichia coli*; BMC, bacterial microcompartment; CCM, CO_2_-concentrating mechanism; cryo-EM, cryo-genic electron microscopy; Rubisco, ribulose-1,5-bisphosphate carboxylase/oxygenase; Raf1, Rubisco assembly factor 1; RbcL, Rubisco large subunit; Prochlorococcus, *Prochlorococcus* MED4.

CBs are highly attractive “plug-and-play” bioengineering targets because they intrinsically exhibit self-assembly, selective permeability, enzyme encapsulation, and structural modularity (Borden and Savage, 2021; Liu et al., 2021). In synthetic biology, one promising strategy is to transplant CBs into heterotrophic microbes such as *Escherichia coli* and yeast (Kerfeld and Sutter, 2020; Correa et al., 2025), thereby constructing novel CO_2_-fixation pathways. Another approach is to introduce CBs into non-native photosynthetic hosts, including plants and algae, to improve photosynthetic efficiency and advance carbon-neutral bioproduction (Raba and Kerfeld, 2022; Nguyen et al., 2025b). In addition to their natural role in CO_2_ assimilation, the modular and programmable protein-based architecture of CB shells offers a versatile platform for the construction of customized nanoreactors or nanocarriers, enabling new catalytic functions, metabolic pathway optimization, and therapeutic delivery applications (Doron and Kerfeld, 2024). To lay the groundwork for rational engineering and realize this potential in synthetic biology and biotechnology, a comprehensive understanding of CB structure, organization, and assembly mechanisms is essential. Because the comprehensive review by Kerfeld and colleagues summarized key developments in BMC research (including CBs) up to 2017 (Kerfeld et al., 2018), this review focuses mainly on advances over the past decade in elucidating CB structure and assembly mechanisms, as well as strategies and efforts to bioengineer CBs for enhanced CO_2_ fixation and novel functions (Figure 2).

## Gene organization and protein stoichiometry

CBs can be classified into two lineages, α-CBs and β-CBs, which differ in the phylogenetic subclass of Rubisco they encapsulate, their protein composition, and their assembly pathways (Cannon et al., 2002; Kerfeld and Melnicki, 2016; Sutter et al., 2021). α-CBs contain form IA Rubisco, whereas β-CBs contain a lineage of the form IB enzyme found in β-cyanobacteria and plants (Rae et al., 2013). α-CBs typically consist of 9–10 polypeptides, including the Rubisco large and small subunits (CbbL/S), carbonic anhydrase (CsoSCA), the linker protein (CsoS2), and a set of shell proteins (CsoS4A/B and CsoS1A/B/C/D/E). Genes encoding α-CB components are usually clustered within a single *cso* operon, whereas β-CB genes are distributed across multiple genomic loci, including a *ccm* operon (Figure 1C). For example, in *Synechococcus elongatus* PCC 7942 (*Syn*7942), a model β-CB system, the *ccm* operon harbors genes encoding the Rubisco large and small subunits (RbcL/S), shell proteins (CcmK2/L/O), and linker proteins (CcmM/N), whereas other CB-associated genes, such as *ccaA* (encoding CA), *ccmK3/K4* (encoding the minor hexameric shell proteins CcmK3/K4), and *ccmP* (encoding the trimeric shell protein CcmP), are located outside the *ccm* operon (Figure 1C). This dispersed gene arrangement may provide greater regulatory flexibility, potentially contributing to the notable variation in protein stoichiometry and diameters of β-CBs in *Syn*7942 under different conditions and suggesting a mechanism for coordinated expression from multiple genomic loci.

Precise protein stoichiometry is fundamental to CB assembly and the successful bioengineering of functional CBs. Recent studies using quantification conCATamer (QconCAT)-based mass spectrometry have revealed the absolute protein stoichiometry of native α-CBs from *Halothiobacillus neapolitanus* C2 (*Halo*) and β-CBs from *Syn*7942 grown under low light (Figure 1D) (Sun et al., 2022, 2025b). These measurements correspond to CBs with the following mean observed structural dimensions: the *Halo* α-CB has a molecular mass of ∼346 MDa and a diameter of ∼120 nm, whereas the *Syn*7942 β-CB is larger, at ∼529 MDa and ∼170 nm, likely representing the largest known proteinaceous complexes. Individual CBs within a population, and under different growth conditions, can nevertheless be considerably smaller or larger than these mean values. In both types of CB, hexameric shell proteins tiling the facets involve ∼975 CsoS1A/B/C copies in the *Halo* α-CB and ∼1998 CcmK2/K3/K4 copies in the *Syn*7942 β-CB. In an ideal CB structural model, 12 pentamers would occupy the 12 vertices of the typical icosahedral architecture. However, α-CBs contain 11 CsoS4 pentamers, and β-CBs contain only 6 CcmL pentamers, indicating that both CB lineages diverge from ideal icosahedral symmetry by incorporating vertex vacancies or structural defects and irregularities, consistent with structural observations (Hagen et al., 2018; Ni et al., 2022, 2023).

Rubisco complexes are densely packaged inside CBs, with 447 copies in each α-CB and 639 copies in each β-CB under low-light conditions in the average observed structures. In contrast, CA is much less abundant, with ∼20 hexamers in α-CBs (Sun et al., 2022; Ng et al., 2025) and 28 hexamers in β-CBs (Sun et al., 2025b), highlighting its high catalytic efficiency in supporting Rubisco activity. Indeed, the *k*_cat_ values of carboxysomal CAs (10^4^−10^5^ s^−1^) (Heinhorst et al., 2006; McGurn et al., 2016) are much higher than those of Rubisco (∼1−10 s^−1^) (Prywes et al., 2025), enabling the active sites of each Rubisco to operate at near-maximal efficiency. Interestingly, Rubisco activases have also been detected in purified CBs, with ∼15 CbbQO complexes per α-CB and ∼3 copies of RbcX per β-CB (Figure 1D). It is worth noting that the protein stoichiometry of CA and Rubisco activases may vary among species. In addition to the canonical Rubisco activase, a Rubisco activase–like cyanobacterial protein, ALC, is broadly distributed across cyanobacteria, localizes to CBs, and exhibits ATPase activity (Lechno-Yossef et al., 2019).

Another major component is the linker protein that bridges the shell and cargo. Based on protein stoichiometries from average observed CB structures, each native *Halo* α-CB contains approximately 440 copies of CsoS2, which exists as two distinct isoforms, CsoS2A and CsoS2B, in a stoichiometric ratio of 1.3:1 (Cai et al., 2015; Sun et al., 2022). Although CsoS2B alone was sufficient to enable the formation of intact CBs or empty shells, CsoS2A alone was not; however, CsoS2A plays an important role in promoting the assembly of larger α-CBs and empty shells (Li et al., 2024a; Oltrogge et al., 2024). The native *Syn*7942 β-CB contains ∼842 copies of CcmM, and its two isoforms, CcmM35 and CcmM58, which are named according to their molecular masses, occur at a ratio of ∼6.5:1 (Sun et al., 2025b). Note that the molecular masses and ratios of the two CcmM isoforms may vary among cyanobacterial species. In contrast to the assembly of α-CBs, the assembly of functional β-CB strictly requires both CcmM35 and CcmM58 at functionally relevant stoichiometries, with a minimum ratio close to 1:1 (Long et al., 2010). β-CB assembly is initiated when CcmM35 nucleates Rubisco condensation; CcmM58 subsequently bridges these Rubisco–CcmM35 complexes to CcmN (∼8 copies), thereby mediating shell–cargo association (Cameron et al., 2013).

Although CB protein stoichiometry can vary among individual organelles, species, and growth conditions, absolute quantification of the *Halo* α-CB and *Syn*7942 β-CB provides mean stoichiometric models for α- and β-CBs, respectively. These datasets offer a valuable quantitative framework for understanding CB organization across systems of similar size and composition and for rationally engineering CBs in heterologous hosts.

## Carboxysome architecture and Rubisco packaging mediated by scaffolding proteins

**Figure 3:**
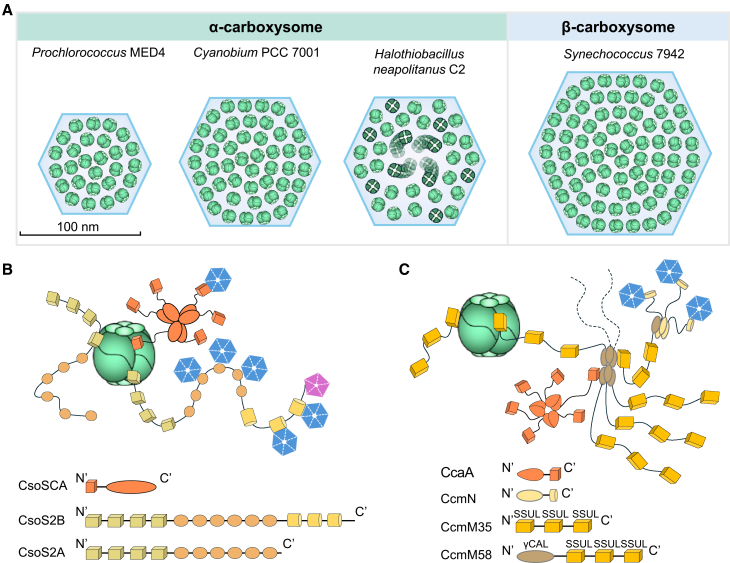
Distinct spatial organization of Rubisco and interaction networks between cargo and linker proteins within α- and β-CBs. **(A)** In α-CBs from α-cyanobacteria such as *Prochlorococcus* MED4 and *Cyanobium* sp. PCC 7001, Rubisco molecules are arranged in concentric layers, whereas in α-CBs from *Halothiobacillus neapolitanus*, Rubisco molecules form intertwined spirals at the center, surrounded by randomly arranged Rubisco particles. Rubisco molecules within β-CBs, such as those from *Synechococcus elongatus* PCC 7942, are also organized in concentric layers. Scale bar, 100 nm. Protein–protein interaction networks formed between linker proteins and cargo enzymes are shown for α-CBs **(B)** and β-CBs **(C)**.

Recent structural studies, particularly those using cryo-electron microscopy (cryo-EM), have substantially advanced our understanding of CB structure, including shell architecture and the internal organization of cargo enzymes. Distinct Rubisco packing patterns have been observed among α-CBs from different species (Figure 3A). In *Halo* α-CBs, along together with randomly distributed ones Rubisco complexes are organized into intertwining spiral arrays in the center of the CB lumen, mediated by electrostatic interactions between the small CbbS subunits of neighboring Rubisco complexes (Metskas et al., 2022; Ni et al., 2022). In contrast, in α-CBs from the cyanobacterial species *Prochlorococcus* MED4 and *Cyanobium* sp. PCC 7001 (*Cyan*7001), Rubisco complexes are organized into three or four concentric layers parallel to the inner shell surface (Figure 3A) (Ni et al., 2022; Evans et al., 2023; Zhou et al., 2024). The latter arrangement resembles that of *Syn*7942 β-CB, in which Rubisco complexes are tightly packed into four to six concentric layers parallel to the shell, with individual Rubisco complexes predominantly oriented in a radial direction extending outward from the center (Figure 3A) (Zhou et al., 2024; Sun et al., 2025b). Whether these distinct Rubisco arrangements contribute to differences in catalytic performance remains an open question. Interestingly, the linker protein CcmM35 has been observed to exhibit dynamic movement within CcmM-mediated Rubisco condensates *in vitro* (Wang et al., 2019), a process that is redox-modulated, whereas Rubisco itself remains largely immobile (Zang et al., 2021). This dynamic behavior of CcmM35 may facilitate the spatial organization of Rubisco during β-CB biogenesis and contribute to the rigid, ordered arrangement of Rubisco in mature β-CBs. Further investigation into the dynamic behavior of both the linker protein and Rubisco *in vivo* will enhance our understanding of the mechanisms that underlie the spatial organization and biogenesis of β-CBs.

In both CB lineages, linker/scaffolding proteins play essential roles in Rubisco encapsulation and in the establishment and maintenance of spatially organized Rubisco networks within CBs. CsoS2 is an intrinsically disordered, multidomain scaffolding protein essential for α-CB assembly (Figure 3B) (Cai et al., 2015; Turnsek et al., 2024). *Halo* CsoS2 comprises three distinct regions: an N-terminal region (CsoS2-N), a middle region (CsoS2-M), and a C-terminal region (CsoS2-C), each of which contains multiple repetitive elements (Chaijarasphong et al., 2016; Li et al., 2024a). CsoS2-N directly recruits form IA Rubisco through repeated binding modules, enabling the multivalent tethering of multiple Rubisco complexes and driving condensation of the enzymatic core (Oltrogge et al., 2020; Ni et al., 2022). Notably, CsoS2 associates uniformly with all Rubisco proteins in *Cyan*7001 α-CBs, whereas in *Halo* α-CBs, CsoS2 binds exclusively to Rubisco located near the shell and is absent from the interior, where Rubisco complexes organize into spiral arrays (Sun et al., 2022). CsoS2-C interacts with shell proteins, functioning as a “molecular thread” that stitches shell components together and likely initiates shell assembly (Figure 3B) (Ni et al., 2023). The CsoS2-M region is involved in regulating α-CB size, as each repeat binds at the junction of three hexameric shell proteins; accordingly, a greater number of repeats in CsoS2-M correlates with larger α-CB size (Li et al., 2024a; Oltrogge et al., 2024). In some species, such as *Halo* and the unicellular cyanobacteria *Synechococcus* sp. WH7805 and *Prochlorococcus marinus* str. MIT9313, a ribosomal frameshift generates two CsoS2 isoforms: full-length CsoS2B and the truncated form CsoS2A, which lacks the C-terminal domain (Chaijarasphong et al., 2016). Structural analysis revealed that CsoS2A cooperates with CsoS2B to organize shell proteins and encapsulate cargo, resulting in larger CBs than those formed by CsoS2B alone (Li et al., 2024a; Oltrogge et al., 2024).

In β-CBs, CcmM and CcmN serve as essential scaffolding proteins that coordinate shell encapsulation and Rubisco organization (Figure 3C) (Sun et al., 2021; Zang et al., 2021). Like CsoS2 in α-CBs, CcmM in β-CBs exists as two isoforms: a full-length isoform and a shorter isoform. The latter is an N-terminally truncated form, in contrast to the shorter CsoS2 isoform, which is C-terminally truncated. Full-length CcmM contains an N-terminal γ-type carbonic anhydrase-like (γCAL) domain followed by multiple Rubisco small subunit-like (SSUL) repeats, whereas the short isoform comprises only the SSUL domains. CcmM functions as a central organizer of the enzymatic core, linking form IB Rubisco and carbonic anhydrase (CcaA) prior to shell assembly (Long et al., 2007; Wang et al., 2019), and the appropriate stoichiometric balance of both CcmM isoforms is essential for β-CB assembly (Long et al., 2010). The γCAL domains interact with the C-terminal tails of CcaA subunits and orchestrate head-to-head associations of CcmM trimers, whereas the SSUL domains form direct contacts with Rubisco at its equatorial surface and simultaneously participate in intermolecular interactions with the γCAL domains (Figure 3C) (Zang et al., 2021). These multivalent interactions promote liquid–liquid phase separation, driving the formation of a Rubisco–CcmM–CA condensate network that establishes the foundation for β-CB biogenesis. CcmN acts as an adaptor protein, bridging the Rubisco–CcmM–CA matrix to the shell through interactions with shell proteins such as CcmK and CcmL (Sun et al., 2021). A recent structural study showed that recombinantly expressed *Syn*7942 CcmN assembled into a tetramer and that three CcmN protomers could form a heterotetramer with a single CcmM monomer when CcmN was co-expressed with CcmM in *E. coli* (Zang et al., 2026). The absence of either CcmM or CcmN disrupts Rubisco organization and prevents proper CB formation (Cameron et al., 2013).

## Carbonic anhydrase structure and encapsulation

CA is a catalytically essential enzyme within CBs, despite being present at substantially lower abundance than Rubisco. Although diverse, independent CA families exist (α, β, γ, δ, θ, η, ζ, and ι), only β- and γ-type CAs have been found to be integrated into CBs. The precise localization of CA in native CBs remains poorly understood, largely because of its low abundance and likely flexible association. In α-CBs, CsoSCA (β-CA) is thought to associate with the inner shell surface, as it can be incorporated into empty shell structures, and the majority of CA activity remained with the shell fraction when CBs were disrupted (So et al., 2004; Li et al., 2020; Huang et al., 2022). Recent cryo-EM studies revealed that both *Halo* CsoSCA and *Cyan*7001 CsoSCA adopt a trimer-of-dimers oligomeric structure (Pulsford et al., 2024; Ng et al., 2025), and *Halo* CsoSCA can bind to the inner surface of a synthetic shell through interactions with the CsoS1A shell protein to ensure its encapsulation (Ng et al., 2025). The N-terminal domain of CsoSCA is sufficient to recruit Rubisco to the shell, confirming that CsoSCA interacts with Rubisco via its N-terminal region (Figure 3B) (Blikstad et al., 2023; Ng et al., 2025). The physical proximity between CsoSCA and Rubisco within the shell likely provides the structural basis for enhancing Rubisco carboxylation by sustaining elevated CO₂ levels around Rubisco. Intriguingly, the binding site for CsoSCA partially coincides with that of the scaffolding protein CsoS2 on Rubisco, indicating the modularity and plasticity of Rubisco’s binding interface (Figure 3B). Furthermore, the CA from *Cyan*7001 is allosterically activated by the Rubisco substrate ribulose-1,5-bisphosphate (RuBP), forming a feedback loop at a key junction in α-cyanobacterial carbon metabolism (Pulsford et al., 2024).

In β-CBs, CcaA from *Synechocystis* sp. PCC 6803 (*Syn*6803) adopts a compact trimer-of-dimers organization in which the dimers are arranged with their longest axes orthogonal to the molecular three-fold axis (McGurn et al., 2016). Notably, unlike its α-CB counterparts, CcaA does not directly interact with Rubisco. Instead, it can be recruited into Rubisco condensates through interactions with the N-terminal domain of CcmM (Figure 3C) (Zang et al., 2021). In *Syn*7942, CcaA content in β-CBs correlates strongly with **the abundance** of the long CcmM isoform, CcmM58: overexpression of CcmM58 increases both its own inclusion and that of CcaA within β-CBs (Long et al., 2011). Interestingly, not all β-CBs possess dedicated β-CAs, as a specific lineage of β-cyanobacteria naturally lacks the *ccaA* gene that encodes β-CAs; in these species, the N-terminal domain of CcmM serves as a functional γ-type CA that undergoes redox regulation (Peña et al., 2010; de Araujo et al., 2014).

## Shell organization and permeability

The proteinaceous shell of CBs ensures the physical encapsulation of the enzymatic core and functions as a semi-permeable barrier to maintain a high-CO_2_ microenvironment, thereby enhancing Rubisco carboxylation activity. Disruption of shell formation abolishes CO_2_ fixation under ambient air, causing cells to depend on elevated CO_2_ concentrations for growth (Price and Badger, 1989; Cameron et al., 2013; Desmarais et al., 2019). The nanoscale shell architecture, with **its intrinsic capacity** for cargo recruitment and permeability, has attracted considerable interest for bioengineering applications.

Structurally, the shell exhibits icosahedral geometry and is constructed from a monolayer of shell protein assemblies comprising hexamers, pentamers, and trimers (Price and Badger, 1989; Kerfeld and Melnicki, 2016). The major building blocks are BMC-H proteins (such as CsoS1A/B/C in α-CB and CcmK1–6 in β-CB), which assemble into flat hexameric tiles that form the facets of the polyhedron. The vertices are sealed by BMC-P pentamers, such as CsoS4A/B in α-CBs and CcmL in β-CBs, whereas BMC-T trimers, such as CsoS1D in α-CB and CcmO/CcmP in β-CB, are minor shell components located within the shell facets.

High-resolution cryo-EM structures of simplified CB assemblies and native CB shells have revealed the intricate protein–protein interactions governing shell formation (Sutter et al., 2019; Tan et al., 2021; Ni et al., 2023; Wang et al., 2024). The amino acid residues at the pentamer–hexamer and hexamer–hexamer interfaces are highly conserved across different BMC types (Sutter et al., 2017, 2019; Ni et al., 2023; Wang et al., 2024; Zhou et al., 2024). Structural analysis of a synthetic α-CB shell, which is 54 nm in diameter, revealed that the pentamer–hexamer interfaces are primarily stabilized by two types of interaction: a salt bridge formed between CsoS4A Asp48 in the GCxPGD motif, which is **analogous to** the BMC-H GAGxGE motif and CsoS1A Arg83 in the (A/P)RPH motif, and hydrogen bonds between CsoS4A Gly43 in the GCxPGD motif and CsoS1A Lys29 in the KAA motif (Wang et al., 2024). The angles at these pentamer–hexamer interfaces are largely consistent, varying only between 29.4° and 31.5°, comparable to those observed in native α-CBs from *Prochlorococcus* MED4 with a diameter of 86 nm (32°) and a synthetic 40-nm BMC shell from *Haliangium ochraceum* (HO shell, 30°) (Sutter et al., 2017; Wang et al., 2024; Zhou et al., 2024). In contrast, the angles at the hexamer–hexamer interfaces exhibit substantial variation, ranging from 0° to 33.6° in synthetic shells and from 0° to 36° in *Prochlorococcus* MED4 α-CBs, with tilt angles gradually decreasing from the pentamer vertices toward the shell periphery. Despite this geometric variability, interactions between adjacent hexamers are stabilized by a conserved hydrogen-bonding network mediated by Lys residues in the KAA motif of one hexamer and by Arg residues in the (A/P)RPH motif of the other hexamer (Sutter et al., 2017; Wang et al., 2024; Zhou et al., 2024). These findings suggest that shell size and overall geometry are governed primarily by the flexibility of hexamer–hexamer interactions, whereas pentamer–hexamer interactions remain structurally rigid.

BMC-T proteins have not been explicitly detected in either synthetic CB shells or native CB structures, possibly because of their low abundance and flexible positioning within the shell. In contrast, the HO shell contains a higher proportion of BMC-T proteins, with a substantially greater BMC-T:BMC-H ratio of 1:3 compared with those of CBs (1:325 for native *Halo* α-CBs and 1:333 for native *Syn*7942 β-CBs) (Sutter et al., 2017; Greber et al., 2019; Sun et al., 2022, 2025b). Notably, structural analysis has revealed that the same Lys residues that mediate hexamer–hexamer contacts also participate in trimer–hexamer interfaces through interactions with conserved Lys residues in BMC-T proteins of HO shells. In addition, contacts between less-conserved residues in the BMC-T C-terminal domain and conserved Arg residues in the (A/P)RPH motif of BMC-H further stabilize trimer–hexamer interfaces (Greber et al., 2019). These interactions, mediated by less-conserved residues in BMC-T proteins, likely contribute to the particularly high abundance of BMC-T proteins in HO shells and thus represent a promising engineering target for enhancing BMC-T incorporation into CBs.

To date, the exact roles and spatial organization of multiple shell protein paralogs in CBs remain unclear. The abundance of individual shell proteins varies in response to environmental factors such as CO_2_ concentration and light intensity, suggesting regulatory adaptation of shell composition (Sun et al., 2019). CcmK3 and CcmK4 from β-CBs have been shown to form pH-dependent heterohexamers (Sommer et al., 2018; Garcia-Alles et al., 2019), indicating a potential role in the dynamic modulation of shell permeability to control metabolite exchange. Although neither CcmK3 nor CcmK4 is essential for CB biogenesis in *Syn*7942 (Rae et al., 2012), deletion of CcmK4 impaired cell growth, indicating that the loss of CcmK4 cannot be functionally **compensated** **by** CcmK3 (Sommer et al., 2018). Similarly, in α-CBs, CsoS1 and CsoS4 paralogs can assemble into heterooligomers, contributing to shell size regulation and architectural flexibility (Wang et al., 2024). The presence of shell protein paralogs likely increases structural flexibility and provides a mechanism for fine-tuning shell permeability and mechanical properties, enabling CBs to adapt to fluctuating environmental conditions.

Each shell protein contains a central pore that varies in size across the three types of shell proteins, functioning as a molecular sieve to regulate metabolite diffusion based on size, charge, and polarity (Tsai et al., 2007; Cai et al., 2009). Previous computational simulations of individual shell hexamers suggested that the positively charged pores of shell hexamers preferentially permit the passage of negatively charged HCO_3_^−^ while restricting the leakage of neutral gases such as CO_2_ and O_2_ (Mahinthichaichan et al., 2018; Faulkner et al., 2020). Recent molecular dynamics simulations of a synthetic β-CB shell suggest that CO_2_ and O_2_ exhibit higher permeability than other negatively charged metabolites, diffusing through both the central pores of shell proteins and the protein–protein interfaces (Sarkar et al., 2024). Nevertheless, CO_2_ leakage occurs at a rate significantly lower than its turnover by Rubisco, owing to rapid CO_2_ generation by CA and the ability of the protein shell to act as a physical barrier that effectively redirects CO_2_ back into the CB lumen when it collides with the shell’s inner surface. In this manner, together with efficient CA activity, the shell effectively fulfills its CO_2_-concentrating role, maintaining elevated CO_2_ levels around Rubisco to enhance carboxylation efficiency while restricting oxygenation activity. BMC-T proteins, which possess larger and potentially gated pores, are thought to undergo conformational changes that regulate the passage of the large metabolites 3-phosphoglycerate and RuBP (Klein et al., 2009; Larsson et al., 2017). Structural analysis of the HO shell showed that stacked BMC-T trimers display conformational flexibility, undergoing “breathing-style” transitions between open and closed pore states, thereby expanding the permeability properties of the shell (Greber et al., 2019).

The CB shell has also been proposed to be permeable to protons (Menon et al., 2010), thereby maintaining pH equilibrium between the CB interior and the cytoplasm. Computational modeling has suggested that a relatively acidic carboxysomal pH (∼7) can enhance CCM efficiency (Mangan et al., 2016). Another modeling study indicated that proton production by Rubisco reactions contributes to the acidic environment inside CBs (Long et al., 2021). Notably, RuBP plays a role in modulating proton concentration in CBs by carrying protons from the exterior to the interior and by allosterically activating CA (such as that from *Cyan*7001), thereby indirectly influencing proton balance (Long et al., 2021; Pulsford et al., 2024). Recent experimental studies using a pH-sensitive GFP variant revealed that the internal pH of α-CBs is lower than that of the surrounding cytoplasm, suggesting the presence of a controlled proton microenvironment (Huang et al., 2022). This mildly acidic interior may enhance CA activity, facilitating the rapid conversion of HCO_3_^−^ into CO_2_. In addition, direct redox measurements within β-CBs using roGFP2 revealed that their lumens are more oxidizing than the surrounding cytosol (Huffine et al., 2026). The more oxidizing environment inside CBs may facilitate CA activation, whereas CA activity is kept low in the relatively reduced cytosol, helping to limit CO_2_ escape from the cell (Peña et al., 2010; de Araujo et al., 2014). This finding suggests that the shell restricts the entry of reducing agents, thereby helping to preserve enzyme activity under fluctuating cellular conditions.

## Carboxysome biogenesis pathways

CB assembly depends on multivalent, phase-separating scaffolds that organize enzymatic cargo and coordinate shell formation, although the specific assembly pathways differ between the two CB lineages (Ang et al., 2023). β-CB biogenesis proceeds through a “core-first” mechanism, in which internal enzymes assemble first before shell formation (Cameron et al., 2013; Chen et al., 2013). During early biogenesis, Rubisco holoenzymes assemble and become tightly packed with the help of dedicated chaperones such as Raf1 and RbcX (Huang et al., 2019, 2020; Xia et al., 2020), while the multivalent scaffolding protein CcmM binds Rubisco, through its repeated SSUL domains, and β-CA, through its N-terminal domain, driving the formation of a liquid-like Rubisco–CcmM–CcaA condensate, or “procarboxysome” core (Wang et al., 2019; Zang et al., 2021). Once this interior matrix has formed, the adaptor protein CcmN binds to CcmM to form heterotetramers in which the SSUL domain of the CcmM protomer associates with the procarboxysome core by interacting with Rubisco and the γCAL domains of CcmM trimers (Figure 3C) (Zang et al., 2026). Moreover, this CcmM–CcmN heterocomplex specifically recruits shell proteins through the CcmN C terminus to build the shell architecture surrounding the enzymatic core (Zang et al., 2026). These findings indicate that Rubisco nucleation and condensate formation are prerequisites for shell recruitment and organelle formation in β-CBs. Recent studies have also revealed that β-CB shell assembly is assisted by external factors in specific species, such as CcmS, identified in *Syn*6803 and *Nostoc* sp. PCC 7120 (Chen et al., 2023b; Cheng et al., 2024). In these organisms, CcmS forms a stable homodimer and interacts with the C-terminal extension of CcmK1, mediating shell assembly and intact β-CB formation. Notably, CcmS, and often CcmK1, are absent from many β-CB operons, indicating that the CcmS–CcmK1 module represents a species-specific chaperone system for shell maturation rather than a universal β-CB assembly factor.

Intriguingly, the core-first assembly pathway of β-CBs differs from the assembly mechanisms of α-CBs and catabolic BMCs in *Salmonella* (Yang et al., 2022, 2025; Chang et al., 2026). Earlier observations suggested that α-CB assembly proceeds through a “shell-first” or coupled shell–cargo mode (Menon et al., 2008; Iancu et al., 2010). Consistent with this scenario, empty α-CB shells can self-assemble independently of cargo (Li et al., 2020). The linker protein CsoS2 orchestrates α-CB assembly by mediating interactions with Rubisco and shell proteins: its N-terminal motifs bind Rubisco through multivalent contacts (Oltrogge et al., 2020; Ni et al., 2022), whereas its C-terminal region interacts with shell components to drive shell formation (Ni et al., 2023). CsoSCA is likely recruited to α-CBs through interactions with both the inner surface of shell proteins and Rubisco. Moreover, Rubisco activation in α-CBs is mediated by the CbbQO complex, which consists of a CbbQ hexamer containing a canonical AAA^+^-ATPase domain and a single CbbO monomer that acts as a Rubisco adaptor (Sutter et al., 2015; Chen et al., 2022).

## Expression of carboxysomes in heterologous hosts to enhance carbon fixation

**Figure 4:**
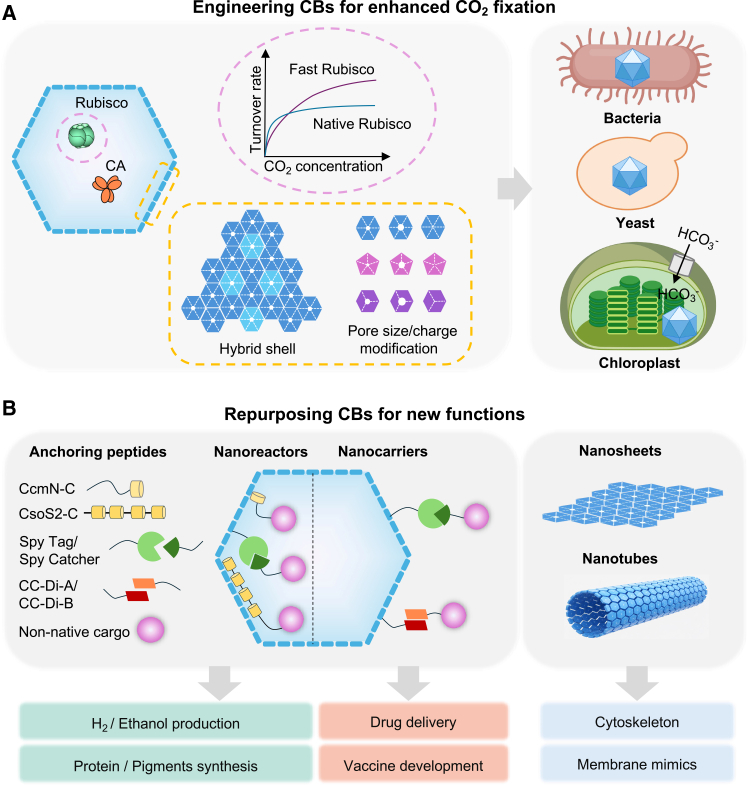
Conceptual map of future applications for engineered CBs. **(A)** CB performance can be improved by replacing native Rubisco with faster or more efficient Rubisco variants to enhance CO_2_ fixation. CB shells can be engineered by incorporating non-native shell paralogs to generate hybrid shells or by redesigning the pore size and charge of shell proteins to achieve the desired permeability for substrate uptake and product release. Native and engineered CBs can be transplanted into industrial microorganisms, such as *E. coli* and yeast, to establish new CO_2_-fixation pathways. Engineering both CBs and inorganic carbon transporters into plants holds promise for increasing photosynthetic CO_2_-fixation efficiency, thereby improving plant growth and biomass accumulation. **(B)** In addition to their use in carbon fixation, CBs can be repurposed as programmable nanoreactors or nanocarriers for customized functions. Non-native cargoes can be encapsulated within the shell via endogenous encapsulation peptides or immobilized on the shell surface using synthetic interaction pairs. Individual BMC-H shell proteins can also self-assemble into higher-order structures, such as nanosheets and nanotubes, enabling their use as protein-based nanomaterials.

Leveraging insights into CB structure and assembly has enabled significant advances in CB engineering and the modification of encapsulated Rubisco to enhance carbon fixation and adaptability in heterologous systems (Figure 4A). Intact and catalytically active α-CBs have been successfully reconstructed in *E. coli* by expressing 10 *Halo* CB genes (*cbbL/S*, *csoS2*, *csoSCA*, *csoS4A/B*, *csoS1A/B/C*, and *csoS1D*) (Bonacci et al., 2012). Further incorporation of the Rubisco activases CbbQ and CbbO into the recombinant α-CBs enhanced Rubisco carboxylation activity (Chen et al., 2022). Attempts to express functional α-CBs have also been made in other biotechnologically relevant hosts, including *Corynebacterium glutamicum* (Baumgart et al., 2017) and *Saccharomyces cerevisiae* (Li et al., 2025). In addition to CB engineering, a functional α-CB-based CCM has been successfully installed in *E. coli*. The expression of 20 CCM genes, including those encoding α-CB proteins and the DAB-type inorganic carbon transporter DabAB1, is sufficient to enable *E. coli* to fix CO_2_ into biomass under ambient air (Flamholz et al., 2020). This platform also provides a foundation for screening various inorganic carbon transporters and identifying potential metabolic bottlenecks through comprehensive metabolomic analysis (Chen et al., 2026).

Native α-CBs sequester form IA Rubisco, whereas β-CBs recruit form IB Rubisco, reflecting their distinct evolutionary lineages and encapsulation mechanisms. Building on this distinction, hybrid CBs have recently been constructed by replacing native form IA Rubisco with more catalytically efficient form IB Rubisco within α-CB shells. For example, form IB Rubisco from *Thermosynechococcus elongatus* BP1 was encapsulated inside recombinant *Cyan*7001 α-CB shells, forming CB-like structures (Nguyen et al., 2023). Likewise, form IB Rubisco from *Syn*7942 was packaged within *Halo* CB shells using a chimeric linker protein in which the N-terminal domain of CsoS2 (the native α-CB scaffold **that recruits** form IA Rubisco) was replaced with the CcmM SSUL domain, the β-CB scaffold that specifically engages form IB Rubisco. This chimeric linker protein substantially enhanced form IB Rubisco encapsulation efficiency compared with native CsoS2, and the resulting hybrid CBs exhibited CO_2_-fixation activity comparable to that of native α-CBs sequestering native *Halo* form IA Rubisco (Sun et al., 2025a). Similar strategies have also been used to generate modular, CB-inspired carbon-fixing compartments by reprogramming encapsulins, a family of bacterial nanocompartments, to sequester diverse Rubisco variants through a short cargo-loading peptide (Szyszka et al., 2025).

By contrast, construction of functional β-CBs has proven more challenging, largely because the molecular determinants that govern β-CB assembly are not fully understood. Early attempts to express native *ccm* operons containing 12 genes, including *rbcL/S*, *ccaA*, *ccmK2–4*, *ccmM*, *ccmL*, *ccmN*, *ccmO*, *ccmP*, and *rbcX*, in *E. coli* resulted in the formation of β-CB-like structures in low yield. This was likely due to improper stoichiometry of β-CB components, as well as the absence of chaperones required for efficient Rubisco core assembly (Fang et al., 2018). Recent studies have demonstrated that co-expression of the assembly factor Raf1 and modulation of RbcL/S stoichiometry greatly improve the assembly of Rubisco and the yield of catalytically active β-CBs in *E. coli* (Sun et al., 2025b). Because cargo nucleation is the first step in β-CB biogenesis, the incorporation of additional assembly factors, such as RbcX, which facilitates Rubisco condensate formation, together with precise control over the ratio of components involved in cargo nucleation (Rubisco, CcaA, CcmM, and CcmN), may further promote β-CB assembly (Huang et al., 2019; Li et al., 2022). Co-expression of auxiliary components such as CcmS, which plays a role in stabilizing shell proteins and assists in shell assembly (Chen et al., 2023b; Cheng et al., 2024), is likely to further improve β-CB assembly efficiency. In addition, recapitulating the native stoichiometry of all β-CB components is expected to be important for robust assembly and maximal catalytic performance in recombinant β-CBs.

Transplanting CBs into C_3_ crops to harness the intrinsically higher catalytic turnover of encapsulated cyanobacterial Rubisco and the locally elevated CO_2_ concentration that enhances its CO_2_-fixation capacity offers a promising strategy for increasing photosynthetic efficiency and, ultimately, crop yields (Hennacy and Jonikas, 2020). Early work demonstrated that specific combinations of β-CB proteins (CcmK2, CcmO, CcmL, and CcmM) can self-assemble into circular shell-like structures within the chloroplast stroma (Lin et al., 2014). Building on this work, Long et al. expressed a minimal gene set (*cbbL*, *cbbS*, *csoS2*, and *csoS1A*) from *Cyan*7001, resulting in the construction of simplified α-CBs in tobacco chloroplasts for compartmentalized CO_2_ fixation under elevated CO_2_ conditions (Long et al., 2018). Furthermore, Chen et al. reported the successful reconstruction of α-CBs containing nine CB components (CbbL, CbbS, CsoSCA, CsoS2, CsoS1A/B/C, and CsoS4A/B) in tobacco chloroplasts (Chen et al., 2023a). Both simplified α-CBs and more fully reconstituted α-CBs expressed in tobacco chloroplasts have been shown to support autotrophic growth and photosynthesis in C_3_ plants under elevated CO_2_. In contrast to simplified α-CBs, which contain only Rubisco, CsoS2, and CsoS1A, the more fully reconstructed α-CBs incorporate additional cargo, including CA, which is crucial for increasing CO_2_ levels near Rubisco to promote carboxylation, as well as additional structural components that are important for proper α-CB assembly and function. Therefore, more fully reconstituted α-CBs may offer advantages for improving functionality in transgenic plants. Collectively, these breakthroughs pave the way for the construction of fully functional CCM modules in plants.

The introduction of CBs alone is insufficient to sustain growth under ambient air owing to the limited HCO_3_^−^ supply within chloroplasts. Establishing a functional CCM requires both the integration of active bicarbonate transporters to ensure adequate HCO_3_^−^ supply and the elimination of endogenous chloroplastic CA to prevent premature conversion of HCO_3_^−^ into CO_2_ outside the CBs (Price and Badger, 1989; Nguyen et al., 2025b). However, stromal CAs play essential roles in maintaining the CO_2_/HCO_3_^−^ balance and pH stability, and their removal may substantially affect carbon assimilation and plant growth. Nevertheless, recent work has successfully eliminated major chloroplast CAs in both *Nicotiana tabacum* and *Arabidopsis thaliana* (Hines et al., 2021; Weerasooriya et al., 2022, 2026), providing suitable platforms for subsequent transporter integration. Incorporation of functional transporters remains challenging, as bacterial bicarbonate transporters such as SbtA and BicA depend on ion gradients and regulatory mechanisms that are absent in chloroplasts (Rottet et al., 2024; Rourke et al., 2026). Functional integration may therefore require matching transporter energetics with chloroplast metabolism and ensuring proper membrane targeting (Rottet et al., 2021). Although bicarbonate channels, such as limited CO_2_-inducible A (LciA) (Guo et al., 2026), could enable passive HCO_3_^−^ diffusion along existing concentration gradients, they **are expected to be** less efficient than active transporters in promoting HCO_3_^−^ accumulation in chloroplasts. Whether these channels can drive HCO_3_^−^ flux into the introduced CBs and are functionally compatible with reconstituted CB-based CCMs in plant chloroplasts remains to be determined. In addition, advances in chloroplast engineering, including enhanced protein expression, coordinated CB assembly, refined shell architecture, and metabolic integration, are essential for installing an operational CCM in plants.

## Repurposing carboxysomes for new functions

Beyond their native role in CO_2_ fixation, CBs have attracted growing interest as highly versatile, proteinaceous scaffolds for the development of modular nanoreactors and nanocarriers. By leveraging their ability to sequester specific biological components, researchers are repurposing these BMCs to host exogenous metabolic pathways and novel catalytic functions (Figure 4B). Compared with β-CBs, which require preassembled native cargo for shell biogenesis, α-CBs exhibit unique structural autonomy. The shell proteins of α-CBs can self-assemble into intact “empty” shells when expressed in heterologous hosts such as *E. coli*. This provides a clean, programmable chassis that can be customized to encapsulate a wide array of non-native enzymes without interference from native metabolic components.

The effective sequestration of foreign cargo within these nanostructures relies on the identification and application of specific native or artificial encapsulation peptides. For α-CBs, the C-terminal domain of CsoS2 has been identified as a primary encapsulation peptide for cargo recruitment (Figure 4B) (Cai et al., 2016; Li et al., 2020; Jiang et al., 2023). This domain facilitates high-affinity interactions between the cargo and the inner surface of the shell proteins. Fusing the C-terminal peptide of CsoS2 to heterologous enzymes can direct the targeted internalization of proteins into α-CBs during assembly. Similarly, the C-terminal domain of CcmN from β-CBs can function as an endogenous encapsulation peptide, directing heterologous cargo into β-CB shells (Cai et al., 2016). Recent advances in synthetic biology have further expanded the potential of α-CBs to recruit proteins and other molecules through the integration of programmable coupling systems. The use of site-directed protein–protein interaction motifs, including SpyTag/SpyCatcher, SnoopyTag/SnoopCatcher, and coiled-coil systems, enables efficient and precise delivery of enzymes, either into the shell lumen or onto its exterior surface, while permitting simultaneous loading of multiple foreign cargo molecules (Veggiani et al., 2016; Hagen et al., 2018; Lee et al., 2018; Kirst et al., 2022; Li et al., 2024b) (Figure 4B). Together, these innovations provide a strong foundation for the precise bioengineering and functionalization of α-CBs to capture, organize, and coordinate biomolecules.

A significant milestone in CB bioengineering is their transformation into specialized nanoreactors for hydrogen production through the encapsulation of hydrogenases. Various hydrogenase-sequestering CB-based nanoreactors have been engineered by replacing native Rubisco with oxygen-sensitive [FeFe]- or [NiFe]-hydrogenases together with their respective electron-transfer partners (Li et al., 2020; Jiang et al., 2023). Shell encapsulation was shown to favor the catalytic activity of hydrogenases and substantially improve the O_2_ tolerance of [FeFe]-hydrogenases. These engineered nanoreactors serve two purposes: they create a localized, concentrated microenvironment for electron transfer, and they potentially provide a physical shield that enhances the oxygen tolerance of hydrogenases compared with their cytosolic counterparts. These nanoreactors exhibited enhanced hydrogen production and greater oxygen tolerance compared with free hydrogenases, providing a valuable proof of concept for the bioinspired design of CB-based structures for sustainable energy production.

The versatility of these systems has been further extended through the development of hybrid systems. A light-driven hybrid nanoreactor was recently created by integrating hydrogen-producing CBs with microporous organic semiconductors (Yang et al., 2024). In this configuration, the semiconductor acts as a synthetic antenna that captures solar energy and transfers the resulting photoexcited electrons to encapsulated hydrogenases within the CB shell. This integration enables the development of a light-driven hydrogen-evolution system that mimics the efficiency of natural photosynthesis while using non-native catalytic pathways. Moreover, the exterior surface of the CB shell provides a high-surface-area scaffold for the organization of complex metabolic cascades. For example, the multienzyme pathway responsible for isopentenyl pyrophosphate biosynthesis has been recruited onto the CB shell surface to enhance metabolic flux toward lycopene synthesis (Zhou et al., 2025).

Furthermore, individual BMC-H proteins can self-assemble into higher-order structures with potential applications as protein-based nanomaterials (Figure 4B) (Young et al., 2017). For example, CcmK1 from *Syn*6803 self-assembles into honeycomb-like nanosheets *in vitro* (Garcia-Alles et al., 2017), and PduA, a BMC-H shell protein from propanediol-utilization BMCs, was shown to form nanofilaments with a diameter of ∼20 nm when expressed alone in *E. coli*. These nanofilaments can serve as scaffolds for the attachment of heterologous cargo proteins through engineered protein–protein interaction motifs (Lee et al., 2018). Overexpression of EutM, a BMC-H protein from ethanolamine-utilization BMCs, also led to the formation of self-assembled nanosheets in *E. coli*. Moreover, enzymes immobilized on EutM-based scaffolds exhibited enhanced stability (Zhang et al., 2018). CcmK2 from β-CBs exhibited the capacity to spontaneously assemble into monodisperse solid microparticles *in vitro*, with highly tunable assembly kinetics, including reversible formation and pH-dependent modulation of particle dimensions (Mak et al., 2025). These studies underscore the remarkable structural plasticity of BMC-H shell proteins and demonstrate their potential as modular scaffolds for engineering programmable, self-assembling biomaterials with customized structural and functional properties.

The natural features of CBs have also inspired the development of CB-mimicking systems that translate these design principles into synthetic platforms. A “primordial CB mimic” was generated by co-encapsulating Rubisco and CA within a lumazine synthase cage to simulate the CO_2_-concentrating function (Frey et al., 2016). Similarly, a CB-inspired electrocatalytic device was engineered by incorporating CA and formate dehydrogenase within a porous electrode, enabling efficient CO_2_ reduction under low-CO_2_ conditions (Cobb et al., 2023). In another approach, hydrogen-bonded organic frameworks were constructed by embedding a Ru-based photocatalyst together with a three-enzyme cascade, achieving continuous CO_2_-to-methanol conversion with high turnover efficiency (Tang et al., 2025). Collectively, these studies illustrate how architectural principles derived from natural CB structures can inform the rational design of next-generation bioinspired nanoreactors for carbon transformation and diverse other applications.

## Challenges and possible solutions

Despite promising advances, several barriers still limit the deployment of engineered CBs in large-scale biotechnological applications. A key challenge in using CBs as bioreactors is our incomplete understanding of shell permeability and metabolite transport in the native context, which constrains the rational design of metabolite flux. Efficient transport of substrates and products, such as HCO_3_^−^, RuBP, and 3-phosphoglycerate, across the shell is essential, as is the supply of ATP to luminal Rubisco activases. Although BMC-H and BMC-T proteins exhibit diverse pore sizes and structures that may enable selective metabolite and cofactor transport, the precise mechanisms merit detailed investigation. Structural studies have provided some insights: the crystal structure of the BMC-H protein CsoS1A from a *Halo* α-CB revealed sulfate ions bound in the hexameric pore, supporting its role in anion transport (Tsai et al., 2007). Similarly, the BMC-T protein CcmP from *Syn*7942 forms a double layer of pseudohexamers with a large central pore and was crystallized in two states: one with glycerol bound in an open pore and another with a larger unidentified ligand in a closed pore, indicating a metabolite-driven gating mechanism (Larsson et al., 2017). In addition to crystallographic studies, computational simulations have contributed substantially to our understanding of shell permeability, although whether these modeling approaches can fully recapitulate the authentic transport dynamics of BMC shells in living cells remains to be experimentally tested. One promising strategy is to engineer specific pore mutations in shell proteins, coupled with the use of tailored molecular probes to directly monitor metabolite flux across the shell. This approach could provide critical insight into the relationship between pore structure and metabolite translocation, enabling the rational design of shell architectures with precisely controlled permeability. In addition, synthetic shells with engineered pores of various sizes and electrostatic properties could be customized to meet the specific requirements of diverse metabolic pathways, enabling efficient encapsulation and turnover of targeted substrates and cofactors. Isolation of intact native CBs would enable direct measurements of shell permeability using *in vitro* approaches such as enzyme-coupled activity assays, fluorescent probe diffusion, and isotope-based flux measurements.

Another major challenge lies in achieving precise control over cargo encapsulation, in terms of both incorporation efficiency and the maintenance of balanced stoichiometry, particularly for multienzyme assemblies. Although endogenous encapsulation peptides and synthetic interaction peptide pairs have been successfully used to target foreign cargo to the shell interior, these strategies have typically resulted in the accommodation of cargo molecules along the inner shell surface, leaving the central lumen largely unutilized. By contrast, native CBs use intrinsically disordered scaffold proteins to organize and fully occupy the shell interior through multivalent interactions with their cognate enzymes. This highly specific natural system provides a blueprint for the *de novo* design of synthetic linker proteins that are capable of recruiting non-native cargo with comparable efficiency, potentially enabling dense and spatially organized cargo loading within engineered shells. Alternatively, the use of multiple yet compatible encapsulation systems may enable more precise multienzyme loading with balanced stoichiometry. In parallel, *in vitro* reconstitution of both shells and their cargo-loading machinery provides a complementary strategy for programmable assembly and fine control over cargo incorporation.

Transplanting CBs into heterologous hosts requires close coordination with host metabolic and regulatory pathways to achieve efficient production of target metabolites. Poor integration can result in inefficient substrate utilization, metabolic burden, and impaired cell growth. Several strategies can be used to overcome these challenges, such as computational modeling to predict pathway conflicts; regulatory circuits to dynamically modulate CB number, positioning, and catalytic activity; and auxiliary systems to enhance metabolite channeling. Adaptive laboratory evolution or targeted engineering of host metabolic networks can also improve compatibility and overall performance. Addressing these bottlenecks will be essential for unlocking the full biotechnological potential of CBs and other BMCs as programmable metabolic nanoreactors.

## Concluding remarks

Recent advances in characterizing the structure, assembly, and functional mechanisms of CBs have substantially advanced our understanding of their central roles in bacterial CCMs and their potential as blueprints for engineering enhanced CO_2_ fixation and novel synthetic functions. However, several challenges must be overcome before their full biotechnological potential can be realized. Key areas that require further exploration include a comprehensive understanding of shell permeability and metabolite transport, precise control of multienzyme cargo encapsulation, and efficient coordination between engineered CBs and host metabolism. Addressing these knowledge gaps will be crucial for constructing functional and efficient synthetic CBs in heterologous systems. Continued progress in structural biology, synthetic biology, and computational modeling will facilitate the rational design of CB-inspired nanocages, nanoreactors, and nanomaterials with customizable morphologies and functions. These advances hold promise for enhancing photosynthetic carbon fixation in plants and microorganisms and establishing CBs as versatile, programmable protein scaffolds for biotechnological applications spanning biosynthesis, biosensing, biocatalysis, and targeted molecular delivery.

## Funding

This work was supported by the 10.13039/501100012166National Key R&D Program of China (2023YFA0914600 and 2021YFA0909600), the 10.13039/501100000268Biotechnology and Biological Sciences Research Council (BB/Y008308/1 and BB/Y01135X/1), the 10.13039/501100000275Leverhulme Trust (RPG-2021-286), and the 10.13039/501100000270Natural Environment Research Council (NE/Z00019X/1).

## Acknowledgments

No conflict of interest is declared.

## Author contributions

T.L. and L.-N.L. conceived and designed the project. T.L. generated the figures with assistance from W.C. and V.C. T.L. and L.-N.L. wrote the manuscript with contributions from all authors.
